# Mucoepidermoid Carcinoma of the Anterior Tongue: A Report of a Rare Case

**DOI:** 10.7759/cureus.79434

**Published:** 2025-02-21

**Authors:** Swapan Purkait, Ananjan Chatterjee, Ishita Banerjee, Moumalini Das, Hiralal Ash, Karthikeyan Ramalingam, Abhishek Banerjee

**Affiliations:** 1 Oral and Maxillofacial Pathology, Buddha Institute of Dental Sciences and Hospital, Patna, IND; 2 Pediatric and Preventive Dentistry, Guru Nanak Institute of Dental Sciences and Research, Kolkata, IND; 3 Oral and Maxillofacial Pathology, Awadh Dental College and Hospital, Jamshedpur, IND; 4 Oral and Maxillofacial Surgery, Buddha Institute of Dental Sciences and Hospital, Patna, IND; 5 Oral Pathology and Microbiology, Malla Reddy Institute of Dental Sciences, Malla Reddy Vishwavidyapeeth, Hyderabad, IND

**Keywords:** carcinoma, head and neck cancer, malignancy, malignant salivary gland tumor, minor salivary gland neoplasm, mucoepidermoid carcinoma, neoplasm, pathology, salivary gland pathology, tongue

## Abstract

Mucoepidermoid carcinoma (MEC) is a commonly observed malignancy of salivary glands. It rarely involves minor salivary glands of the tongue. Most cases are reported in the base and dorsum of the tongue. This report discusses a 62-year-old male patient presenting with a non-healing ulcer and pain on the inferior surface of the tongue. Clinical examination revealed an ulceroproliferative, firm, and exophytic growth on the ventral surface of the anterior tongue. An incisional biopsy and histopathological analysis confirmed a high-grade MEC. The patient underwent surgical excision combined with radiotherapy, followed by long-term monitoring for prognosis assessment.

## Introduction

Mucoepidermoid carcinoma (MEC) is the second most common malignant neoplasm of the salivary glands, accounting for approximately 5% of all head and neck cancers. Among salivary gland tumors, 10-15% are malignant [1,2]. MEC is the second most prevalent tumor of the minor salivary glands, comprising 12-40% of cases worldwide. It commonly affects the parotid gland, particularly in women, and typically occurs in the fifth decade of life. However, when involving minor salivary glands, it is most frequently found on the palate [1,3,4].

MEC is the most common malignant salivary gland tumor of epithelial origin in pediatric and adolescent patients. Other forms of minor salivary gland adenocarcinomas are rarely reported in this age group, with most malignant neoplasms occurring in the parotid gland [4-6]. Approximately 20-50% of the tumors that affect the major and minor glands, respectively, are malignant [4]. The global annual incidence of malignant salivary gland neoplasms ranges from 0.9 to 2.6 cases per 100,000 individuals [6]. In this case report, we present a malignancy involving the minor salivary glands of the tongue in a 62-year-old patient.

## Case presentation

A 62-year-old male patient presented with a persistent non-healing ulcer and pain on the inferior surface of the tongue for four months. The ulcer progressively enlarged, leading to severe pain and difficulty in speech. The patient had a history of chronic tobacco and alcohol use for 40 years. His past medical history, surgical history, and dental history were non-contributory.

The patient first sought consultation from a private dental practitioner at Guru Nanak Institute of Dental Sciences in Kolkata, India. After identifying the lesion, the patient was referred to the Buddha Institute of Dental Sciences in Patna, India, for further assessment. Subsequently, the biopsy was sent to Awadh Dental College, India, and Dr. Karthikeyan Ramalingam for an expert opinion regarding the diagnosis and treatment plan.

**Figure 1:**
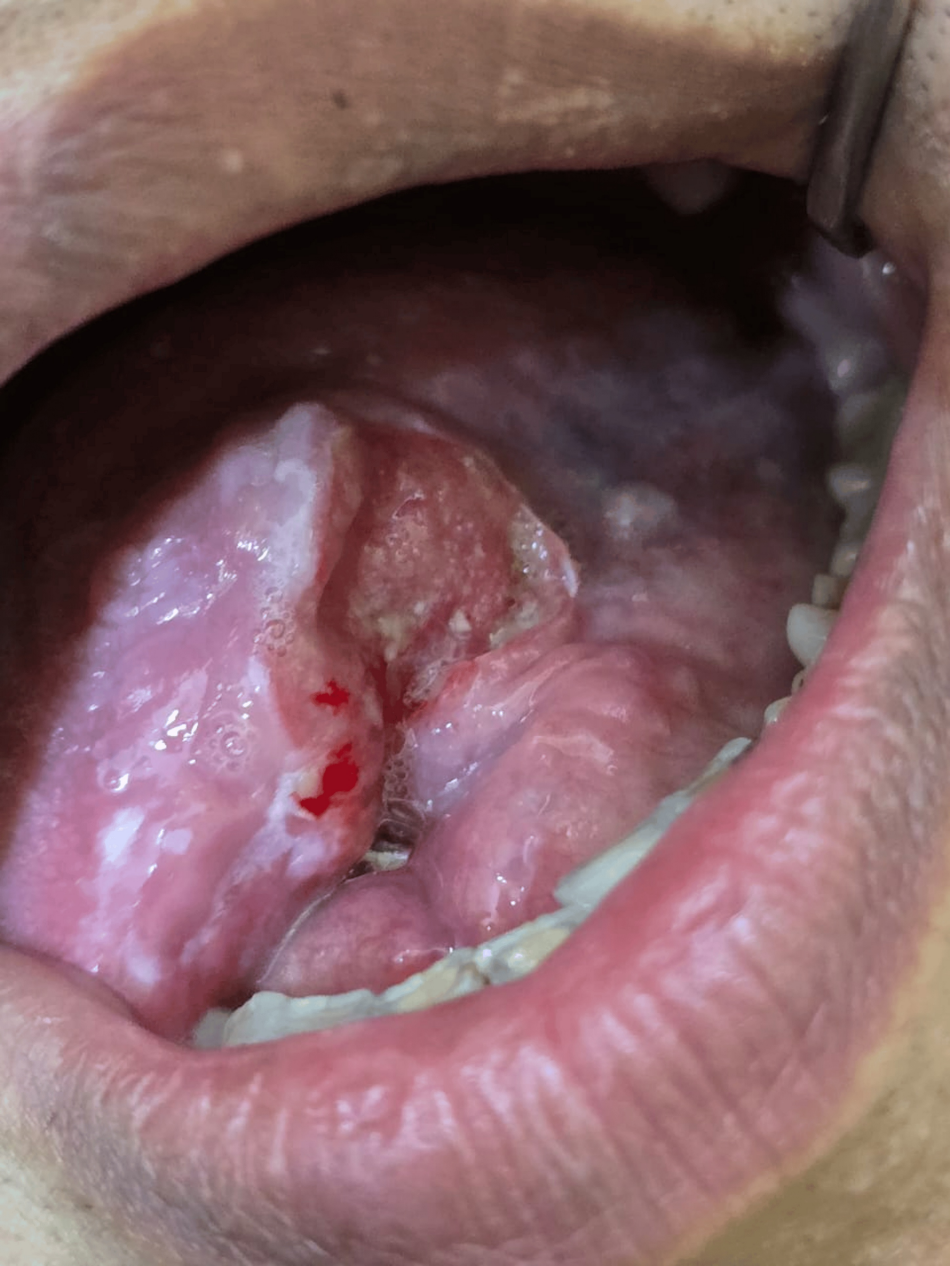
Clinical presentation showing ulceroproliferative exophytic growth on the ventral surface of the anterior tongue

Extra-oral examination did not reveal any abnormalities. Clinical examination revealed an ulceroproliferative, exophytic growth on the ventral surface of the anterior tongue, measuring approximately 6 cm in its largest dimension, with irregular borders (Figure 1). It was firm in consistency and fixed to the underlying muscle. Severe tenderness was noted in the sublingual area on palpation. An incisional biopsy was performed under local anesthesia.

**Figure 2:**
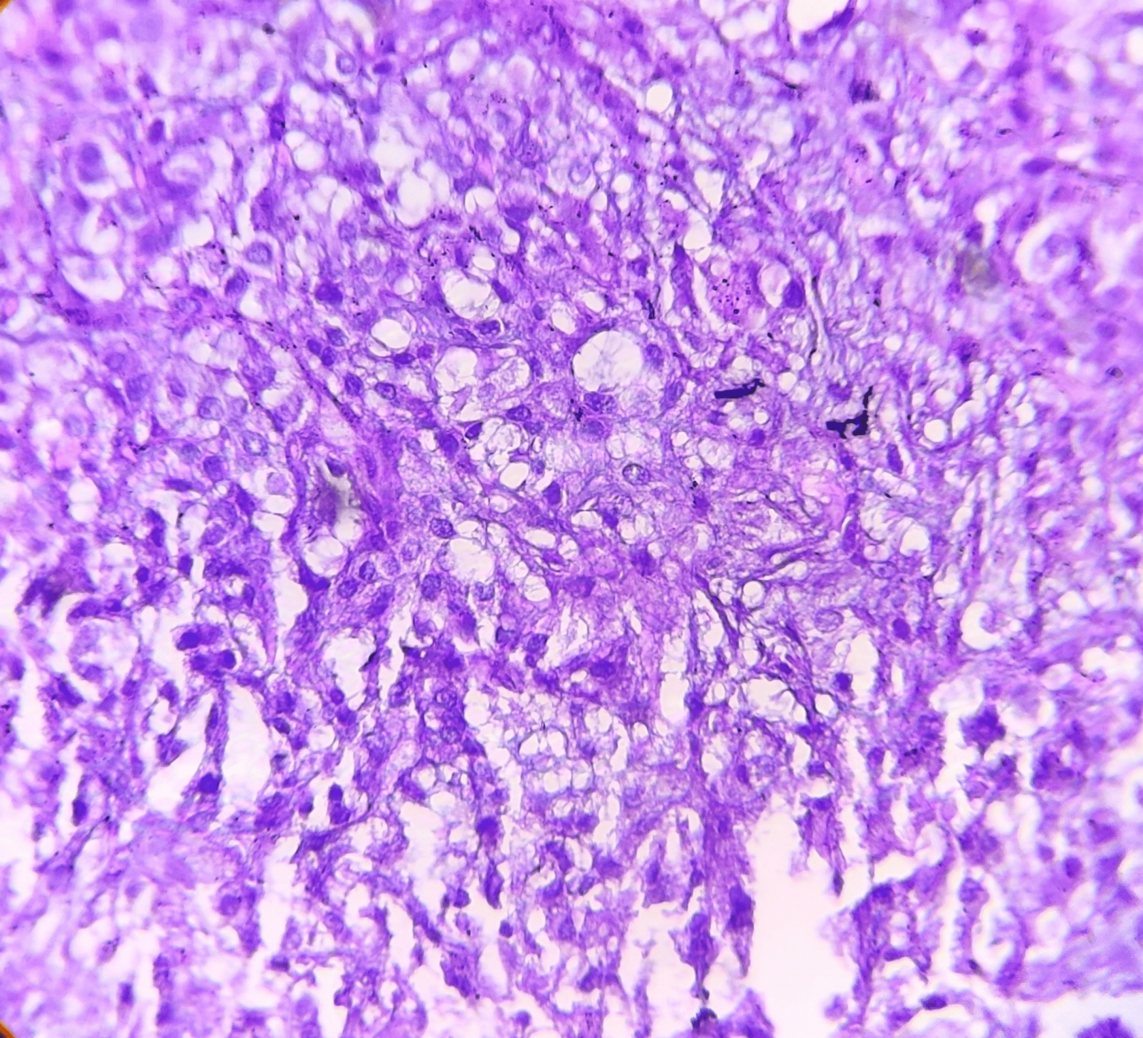
Photomicrograph showing large polygonal epidermoid cells with basophilic cytoplasm (H&E, 20x)

**Figure 3:**
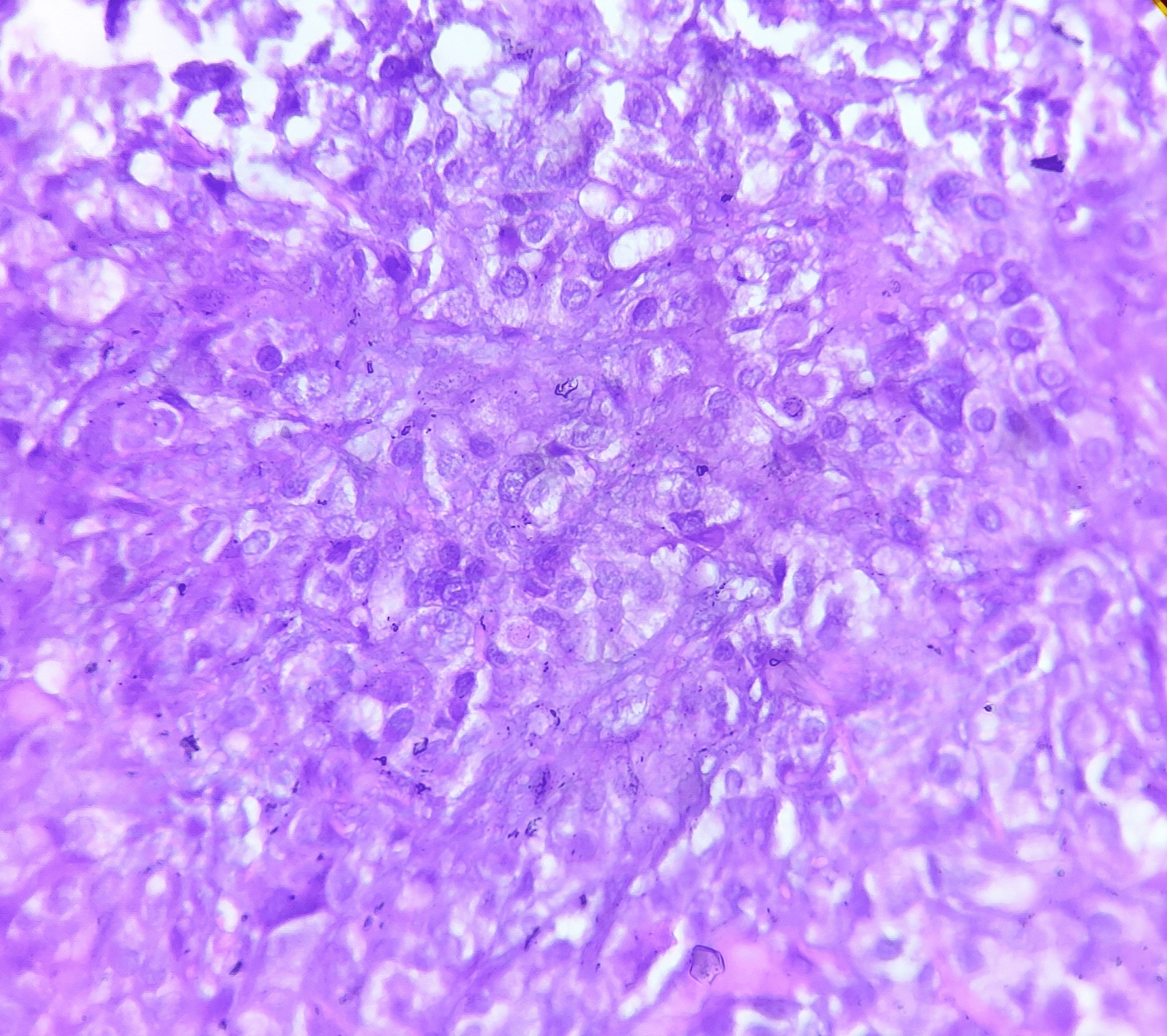
Phtomicrograph showing a mixture of small hyperchromatic abundant intermediate cells with scanty cytoplasm and pale staining mucous cells (H&E, 20x)

Microscopic examination revealed ulcerated squamous epithelium with an intact basement membrane. The connective tissue stroma contained a few islands of large, pale-staining mucous cells, abundant epidermoid cells, and small hyperchromatic intermediate cells. The surrounding stroma consisted of collagen fibers, areas of hemorrhage, and mild chronic inflammatory infiltrate (Figures 2-3).

**Figure 4:**
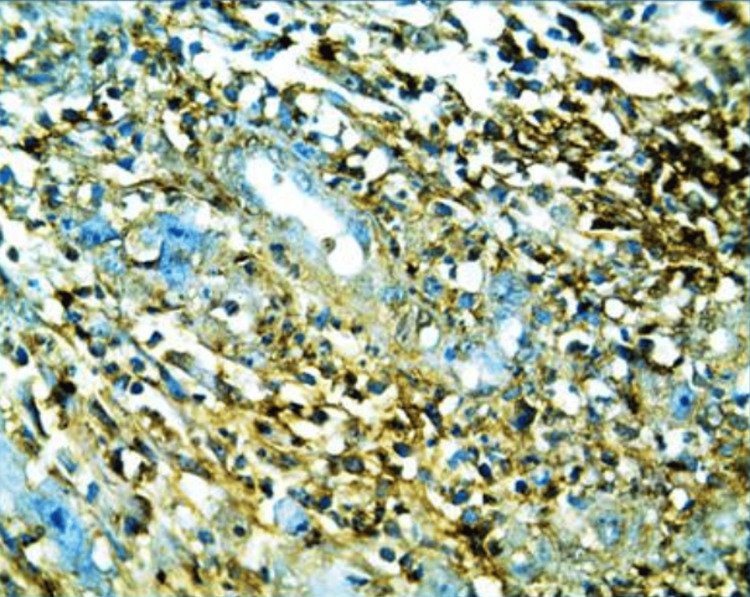
Photomicrograph showing membrane positivity of tumor cells with MUC1 immunohistochemistry (IHC, 20x) IHC: immunohistochemistry

To confirm the salivary gland origin and rule out the clear cell variant of squamous cell carcinoma, immunohistochemical markers cytokeratin 5, cytokeratin 7, and MUC-1 were analyzed. The reports showed positivity to all of these markers (Figure 4).

Based on these findings, a final diagnosis of high-grade MEC was established.

This rare case of MEC was located on the anterolateral border of the ventral surface of the tongue. The patient underwent partial glossectomy with supraomohyoid neck dissection followed by primary closure. A postoperative radiotherapy dose of 60 Gy was delivered via conventional fractionation with photons and electrons using a linear accelerator. Following surgery and radiotherapy, the patient remained stable and was under regular follow-up every six weeks.

## Discussion

MEC is the most common malignant salivary gland tumor, frequently affecting the parotid and minor salivary glands in adults. Although MEC accounts for 30% of all salivary gland malignancies, it represents only 10% of overall salivary gland tumors and less than 5% of head and neck cancers [1,3,5]. Approximately 50-60% of MEC cases originate in major salivary glands, with the parotid gland accounting for over 80% of cases, the submandibular gland 8-13%, and the sublingual gland 2-4%. Minor salivary gland MEC primarily affects the palate, with less common occurrences in the tongue, retromolar region, and buccal mucosa [1,6,7].

MEC most commonly presents in adults aged 40-60 years, with a slight female predominance. Patients often exhibit a slow-growing, painless mass that may be mistaken for a benign lesion, such as a pleomorphic adenoma. However, high-grade MEC is more aggressive, rapidly growing, and often associated with pain, facial nerve involvement, and distant metastases [2,5,8,9]. MECs of the minor salivary glands, especially in the sublingual gland, are frequently malignant, with 70-90% of sublingual tumors classified as malignant. High-grade MECs exhibit rapid growth, local invasion, and a higher risk of distant metastases, particularly to the lymph nodes, bones, and lungs [2,10-12].

Early diagnosis is critical, as clinical and radiographic findings mimic cystic or benign tumors. Histopathological evaluation remains the primary prognostic indicator [2,8,12]. Low-grade MECs typically exhibit a slow-growing, painless nature, while high-grade tumors are more aggressive, characterized by cellular atypia, a lower proportion of mucous cells, and increased metastatic potential [10,13,14]. Treatment primarily involves surgical excision, with the extent of resection determined by tumor location, size, and histological grade. The diagnosis of MECs is mainly based on histopathological assessment with a combination of both H&E and immunohistochemistry tests, such as cytokeratin 5/6 (CK5/6) and transformation-related protein (p63) staining [4]. Our case was diagnosed with histopathology.

While low-grade tumors may be treated with localized resection, high-grade tumors require extensive resection involving adjacent structures [4,10,14]. Neck dissection is recommended in cases of lymph node metastasis. Radiotherapy is generally reserved for high-grade or incompletely excised tumors. Chemotherapy is typically considered for cases with aggressive local or metastatic disease that do not respond to surgery or radiation [12-14]. Literature reports treatment planning based on histopathological scoring of intracystic component, neural invasion, necrotic foci, a mean of ≥4 mitoses per 10 high-power fields, and anaplasia [6]. Prognosis is influenced by tumor grade, site, clinical stage, and adequacy of treatment. Low-grade MECs have a five-year survival rate of up to 95%, while high-grade MECs have a significantly lower survival rate, ranging from 0% to 43% [6,8,14,15]. A total of 12774 cases of minor salivary gland malignancies were found, of which overall MEC prevalence was 16.5% in the systematic review. The retromolar area presented the highest pooled prevalence (58.9%; 95% CI = 47.0%-70.3%), followed by gingiva (28.8%; 95% CI = 22.7%-35.4%) and tongue (27.2%; 95% CI = 21.2%-33.6%) [15]. Our case of MEC involved the anterior tongue. Studies have reported better outcomes in patients undergoing surgical treatment with adjuvant radiotherapy compared to those treated with surgery alone [14,15].

## Conclusions

High-grade MEC is an aggressive malignancy requiring a multidisciplinary treatment approach combining surgery, radiotherapy, and, in some cases, chemotherapy. Early diagnosis through histopathological evaluation is crucial for effective management. The rare anatomical location of this case, along with the patient’s history of tobacco usage, may lead to a diagnostic challenge for both oral pathologists and surgeons. Given the moderate prognosis associated with high-grade MEC, long-term follow-up is essential to monitor for recurrence and metastasis.
